# Seed Nutrition and Quality, Seed Coat Boron and Lignin Are Influenced by Delayed Harvest in Exotically-Derived Soybean Breeding Lines under High Heat

**DOI:** 10.3389/fpls.2017.01563

**Published:** 2017-09-22

**Authors:** Nacer Bellaloui, James R. Smith, Alemu Mengistu

**Affiliations:** ^1^Crop Genetics Research Unit, USDA, Agricultural Research Service Stoneville, MS, United States; ^2^Crop Genetics Research Unit, USDA, Agricultural Research Service Jackson, TN, United States

**Keywords:** soybean germinability, seed quality, seed nutrition, seed coat, boron, lignin, seed protein, seed oil

## Abstract

The timing of harvest is a major factor affecting seed quality in soybean, particularly in Midsouthern USA, when rain during harvest period is not uncommon. The objective of this research was to evaluate the effects of time of harvest on soybean seed quality (seed composition, germination, seed coat boron, and lignin) in high germinability (HG) breeding lines (50% exotic) developed under high heat. The hypothesis was that seeds of HG lines possess physiological and genetic traits for a better seed quality at harvest maturity and delayed harvest. A 2-year field experiment was conducted under irrigated conditions. Results showed that, at harvest maturity, the exotic HG lines had higher seed protein, oleic acid, sugars, seed coat boron, and seed coat lignin, but lower seed oil compared with the non-exotic checks (Control), confirming our hypothesis. At 28 days after harvest maturity (delayed harvest), the content of seed protein, oleic acid, sugars, seed coat boron, and seed coat lignin were higher in some of the HG lines compared with the checks, indicating a possible involvement of these seed constituents, especially seed coat boron and seed coat lignin, in maintaining seed coat integrity and protecting seed coat against physical damage. Highly significant positive correlations were found between germination and seed protein, oleic acid, sugars, and seed coat boron and seed coat lignin. Highly significant negative correlation was found between germination and oil, linoleic acid, seed coat wrinkling, shattering, and hard seed. Yields of some HG lines were competitive with checks. This research demonstrated that time of harvesting is an important factor influencing seed protein and oil production. Also, since high oleic acid is desirable for oxidative stability, shelf-life and biodiesel properties, using HG lines could positively influence these important traits. This result should suggest to breeders of some of the advantages of selecting for high seed coat boron and lignin, and inform growers of the importance of timely harvest for maintaining high seed quality.

## Introduction

Soybean is an important crop in the world and is mainly produced in the USA (36.7%), Brazil (31%), Argentina (16%), and China (4.8%) (Belewu and Belewu, 2007; FAOSTAT, 2011). Seed of soybean is a source of soymeal for livestock, and oil for human consumption (Wilson, 2004), and recently, for biodiesel production (Baldoni et al., 2013; Santos et al., 2013). Soybean seed contain about 37–45% protein, 19 to 25% triglyceride oil composed of various fatty acids (palmitic: 9–14%; stearic: 2–5%; oleic: 19–28%; linoleic: 48–58%; and linolenic 5–11%) (Wilson, 2004; Bellaloui et al., 2017). Soybean seed also contains sugars, such as sucrose, raffinose, stachyose, glucose, and fructose; mineral nutrients including K, Ca, Fe, Mg, Mn, Cu, Zn, and B; and phenolics such lignin and isoflavones (Sakthivelu et al., 2008; Bellaloui et al., 2009, 2010a,b, 2011). High seed protein and oil are desirable for human and livestock nutrition; high oleic and palmitic acids and low linolenic fatty acid are desirable for biodiesel and shelf-life due to oxidative stability of the oil (Ferrari et al., 2005; Atabania et al., 2012; Santos et al., 2013).

In the Midsouthern USA, especially in the Mississippi River Delta region, the Early Soybean Production System (ESPS) led to higher yield under irrigation and non-irrigation; however, the use of this system results in poor seed quality due to high heat, diseases, and early rain during harvest, leading to weathering effects (TeKrony et al., 1996; Mengistu and Heatherly, 2006; Smith et al., 2008; Bellaloui et al., 2012b,c,a). High temperature and delayed harvest, due to rain during harvest, is normal in ESPS, and these conditions lead to seed coat wrinkling and cracking, declining of seed coat physiological integrity, increasing seed deterioration and reducing seed quality (Franca-Neto et al., 1988, 1993).

Two major seed quality components have been reported to be associated with seed germination and seed quality: boron and seed coat lignin. Boron is a micronutrient essential for growth, development, yield and seed quality (Pilbeam and Kirkby, 1983; Marschner, 1995; Brown et al., 1999; Dordas, 2006; Dordas et al., 2007). Boron is also involved in cell wall structure and cell membrane integrity (Hu and Brown, 1994; Brown et al., 2002), nitrogen metabolism (Shelp, 1993; Marschner, 1995), carbohydrate metabolism (Marschner, 1995), including sugar alcohols (Bellaloui et al., 1999; Brown et al., 1999), phenolic metabolism (Marschner, 1995), and nutrient uptake (Goldbach, 1985; Marschner, 1995). Lignin is a major factor influencing seed quality, including germination, shattering, hard-seed, water permeability, and resistance to seed deterioration. For example, de Oliveira et al. (2014) concluded that higher lignin content in the pod and in the seeds may contribute to the decrease in seed deterioration from moisture. Alvarez et al. (1997) observed similar results among the cultivars, with the greatest lignin content in the seed coat for the seeds of the soybean cultivars. However, they also found a significant positive correlation between lignin content in the seed and stink bug damage, indicating that the lignin content in the pod or seed coat did not affect the pod walls resistance to insect.

Previous research showed that seed coat lignin (Krzyzanowski et al., 2008; de Oliveira et al., 2014) and seed B, seed protein, and seed oleic fatty acid, were associated with high seed quality (seed germination and vigor) (Bellaloui et al., 2017), but in those studies delaying harvest was not studied. Time of harvesting is a crop management factor that can significantly affect seed quality (Wilcox et al., 1974; Woodstock et al., 1985) and composition (Woodstock et al., 1985). Adverse weather conditions, such as frequent rain events prevent farmers from harvesting soybeans in the Midsouth USA or tropical regions (de Oliveira et al., 2014) on time, leading to seed quality deterioration (Jaureguy et al., 2013). So far, there are no cultivars with high germinability (HG) or heat tolerance available in the market, although the recent studies of Chebrolu et al. (2016) and Bellaloui et al. (2017) observed new exotically-derived breeding lines with improved seed quality under high heat environments. Therefore, developing breeding lines with high seed quality under high heat conditions of the Midsouthern USA, and understanding the physiology and genetic traits of seed quality responses to delayed harvest is critical and it is the focus of this research. The objective of this research was to evaluate exotically-derived soybean breeding lines with HG for their nutritional qualities and investigate the effects of delayed harvest on soybean seed nutrition quality components (seed chemical composition: protein, oil, fatty acids, sugars; seed coat B and lignin; and seed germination, hard seed, and shattering). To our knowledge, this is the first research to use exotic soybean breeding lines selected for high seed germinability and seed chemical nutrition in such a study. Since boron is known to be involved in seed germination and quality and has a structural role in cell walls and cell membrane (Marschner, 1995; Bellaloui and Brown, 1998), and involved in seed coat integrity and phenolics metabolism (Marschner, 1995), seed coat B and seed coat lignin was also given special attention.

## Materials and methods

### Description of experimental breeding lines

The four exotically-derived breeding lines, two exotic accessions, and six non-exotic checks (four cultivars and two released germplasm lines) evaluated in this study are shown in Table 1. Breeding lines 25-1-1-1-1-4, 30-1-4-1-1, and 34-3-1-2-4-1 were derived from PI 587982A, which was identified by Smith et al. (2008) to have HG. The other parents for each of the above three lines were DT98-9102 (released by USDA-ARS in December 2004, but not entered into GRIN), “5601T” (Pantalone et al., 2003), and DT97-4290 (Paris et al., 2006), respectively. Breeding line 24-2-1-2-1-2 was derived from DT98-9102 × PI603756. The PI 603756, with HG, was also identified by Smith et al. (2008). Each line of the above breeding lines has 50% exotic parentage. One public cultivar from Illinois LD00-3309 (Diers et al., 2006) was included in the study; and two commercial cultivars (AG4903 developed by the Monsanto Corporation and 94B73 developed by DuPont/Pioneer) were also included. It should be noted that all parents of the four breeding lines were included in this study (5601T, DT97-4290, PI587982A, and PI603756). The genotypes used in the study were categorized into two groups: 1- breeding lines derived from exotic parental accessions and previously identified to have HG under irrigation in the ESPS (25-1-1-1-1-4, 34-3-1-2-4-1, 30-1-4-1-1, and 24-2-1-2-1-2) and their exotic parents (PI587982A and PI603756); and 2- cultivars (LD00-3309, 5601T, AG4903, and 94B73) and released germplasm lines (DT97-4290 and DT98-9102).

**Table 1:** Maturity, germination, and yield of genotypes group by their levels of exotic parentage grown under high heat of Mississippi Delta conditions in a 2-year field experiment to test for the effects of delayed harvesting/time of harvest on seed nutrition and quality.

**Genotype**	**Maturity group**	**Days to full maturity (R8)**		**Germination (%)**		**Yield (kg ha^−1^)**	
		**2010**	**2011**	**2010**	**2011**	**2010**	**2011**
		**Days**	**Days**				
**HG (EXOTIC)**							
34-3-1-2-4-1	III	133	146	92.7	92.0	3,306	2,825
30-1-4-1-1	III	119	142	96.0	93.0	2,724	2,219
25-1-1-1-1-4	IV	152	164	92.7	91.0	3,934	2,914
24-2-1-2-1-2	IV	154	164	83.7	90.0	4,006	3,383
PI603756	II	103	119	86.0	95.7	1,222	444
PI587982A	III	122	140	95.7	81.3	1,840	1,446
**CHECKS**							
LD00-3309	IV	133	146	37.3	78.0	3,347	3,268
DT98-9102	IV	165	166	88.7	78.3	3,183	3,051
DT97-4290	IV	161	166	40.7	87.7	3,930	3,697
AG4903	IV	161	166	44.0	87.3	4,528	3,376
94B73	IV	142	154	56.7	76.3	3,901	4,566
5601T	V	172	174	75.7	46.0	3,136	749
LSD				3.7	10.8	221	97

### Field management and growth conditions

A 2-year field experiment was conducted in 2010 and 2011, and planting dates were on 6 April. The experiments were conducted at the Jamie Whitten Delta States Research Center in Stoneville, MS, USA. Each experiment unit was a 4-row plots with a 0.91 m row spacing, and plots were 5.79 m long at planting. The plots were trimmed to 4.88 m long after R1 (beginning bloom and before R6 (full seed) (Fehr and Caviness, 1977). The middle two rows of each plot were harvested with a combine (Almaco, IL, USA) shortly after R8 (full maturity) and weighed for seed yield. We used the term “harvest maturity” to refer to the time of harvest shortly after R8. Fourteen days after the initial harvest of each plot, one border row was hand-harvested and threshed with a bundle thresher. Twenty-eight days after the initial harvest of each plot, the second border row was hand-harvested and threshed as before. Harvested seed was stored at 21°C and 60% relative humidity until assayed for seed quality components. Seed characteristics (composition, seed vigor, hardseededness, and germinability) were made on each replicate. Although the maturity groups (MG) ranged from II to V, the field was designed as such all plots were harvested by the combine as they matured. All plots were furrow irrigated every 7–10 days throughout the growing season as needed to avoid any potential drought stress. After R8 and before initial harvest, plant height, shattering, and lodging were recorded. Physical and chemical analyses indicated that the soil was clay and had adequate soil nutrients. Boron concentration in soil was 2.5 mg kg^−1^, indicating that there was no boron deficiency in the soil. Leaves did not show any nutrient deficiency symptoms.

### Seed analysis for protein, oil, fatty acids, and sucrose

Samples from mature seeds at initial harvest (harvested shortly after full maturity; H1; we have referred to as harvest maturity) and at delayed harvests (14 and 28 days after the initial harvest; H2 and H3, respectively) were analyzed for seed protein, oil, fatty acids, and sugars. About 25 g from each seed sample were ground by a Laboratory Mill 3600 (Perten, Springfield, IL), and protein, oil, fatty acids were analyzed by near infrared reflectance (Wilcox and Shibles, 2001) using a diode array feed analyzer AD 7200 (Perten, Springfield, IL) as detailed by Bellaloui et al. (2009) and Bellaloui et al. (2017). The analysis of the samples was performed based on the reference samples according to AOAC methods for protein and oil. The content of protein in seeds was estimated by measuring total nitrogen using the Kjeldahl method. Protein was calculated from total nitrogen using Dumas, Nx6.25 (AOAC, 1990a). For oil content in seeds, the oil was determined using the Soxhlet extraction method (AOAC, 1990b). Fatty acid analysis was based on reference samples determined by gas chromatography using fatty acids methyl esters (FAME) method (AOAC, 2000). The preparation and analysis of FAME were conducted by direct esterification with methanolic soldium methoxide. The separation was conducted by capillary gas chromatography, and the identification was determined by comparing the samples to pure standards. Calibrations were developed by the University of Minnesota, using Perten's Thermo Galactic Grams PLS IQ software, and established according to AOAC methods (AOAC, 1990a,b) and AOAC (2000). Measurement of protein and oil were reported as a percentage of seed dry matter basis (Wilcox and Shibles, 2001; Boydak et al., 2002) and fatty acids were based on total oil.

For sucrose a similar procedure of seed grinding was followed as indicated above. Briefly, about 25 g of seed from each replicate were ground by a Laboratory Mill 3600 (Perten, Springfield, IL). Seed sucrose content was determined by NIR (Wilcox and Shibles, 2001; Bellaloui et al., 2010b). The calibration equation was developed by the Department of Agronomy and Plant Genetics, University of Minnesota St Paul, MN, using Thermo Galactic Grams PLS IQ software, and developed by Perten company (Perten, Springfield, IL) based on methods of Biermann and McGinnis (1989) and Churms et al. (1982). This method was based on the conversion of sucrose to fructose and glucose using enzyme Invertase (β-fructosidase), and then the glucose produced measured by the hexokinase methods (Biermann and McGinnis, 1989). Sucrose measurement was based on dry matter basis (Wilcox and Shibles, 2001; Boydak et al., 2002; Bellaloui et al., 2010b).

### Seed glucose and fructose analysis

Glucose and fructose were determined in mature seed samples ground by a Laboratory Mill 3600 (Perten, Springfield, IL) to achieve particles uniformity. The Glucose Assay Reagent and sample blanks were prepared as recommended by the manufacturer and as detailed elsewhere by Bellaloui et al. (2014). The absorbance was read at 340 nm using a Beckman Coulter DU 800 spectrophotometer (Fullerton, CA). The concentration of glucose was expressed as mg g dwt^−1^ and the concentration of fructose was measured as mg g dwt^−1^.

### Total boron and seed coat boron analysis

Total and seed coat B were measured in mature seed at each harvesting times [(0, 14, and 28 days after harvest maturity (H1, H2, and H3)]. In case of seed coat B, the seeds were immersed in water for 12 h, the seed coat was removed from the cotyledon, and the seed coat was dried in an oven for 16 h at 105°C. Total and seed coat B were measured according to the Azomethine-H method (Lohse, 1982), and as previously detailed elsewhere (Dordas, 2006; Dordas et al., 2007; Bellaloui et al., 2014). Briefly, the total B was measured in a 1.0-g ground seed sample. The sample was ashed at 500°C, then extracted with 20 ml of 2 M HCl at 90°C for 10 min, and then filtered. Then, a 2-ml sample of the solution was added to 4 ml of buffer solution, containing 25% ammonium acetate, 1.5% EDTA, and 12.5% acetic acid and 4 ml of freshly prepared azomethine-H solution (0.45% azomethine-H and 1% of ascorbic acid) (John et al., 1975). After at least 45 min of color development, B concentration was determined in the samples using a Beckman Coulter DU 800 spectrophotometer (Fullerton, CA) at 420 nm.

### Seed coat lignin

The content of seed coat lignin of mature seed at different harvesting times was determined according to Alvarez et al. (1997) and Krzyzanowski et al. (2001). After the seeds were immersed in water for 12 h, the seed coat was removed from the cotyledon, and the seed coat was dried in an oven for 16 h at 105°C. Then, the samples were removed and placed in a desiccator at room temperature. Then, 250 mg of dry seed coat were homogenized with a mortar and pestle in 7 ml of 50 mM potassium phosphate buffer at pH 7.0 and the mixture was transferred into a centrifuge tube (Ferrarese et al., 2002) and centrifuged at 1,400 g for 10 min. The pellet received three consecutive washes with different chemical solutions (twice with 7 ml phosphate buffer pH 7.0; three times with 7 ml 1% (v/v) Triton X-100 in pH 7.0 buffer; two times with 7 ml 1 M NaCl in pH 7.0 buffer; two times with 7 ml with distilled water; and two times with 5 ml acetone). The pellet was dried in an oven then placed into a vacuum desiccator to cool. This dry matter was considered as a protein-free cell wall fraction. A volume of 1.2 ml thioglycolic acid with 6 ml 2 M HCl was added to the dry matter and heated at 95°C for 4 h. After cooling, the mixture was centrifuged at 1,400 g for 5 min, and the supernatant was discarded. The pellet containing the lignin–thioglycolic acid (LTGA) complex was washed with distilled water, and the LTGA was extracted by shaking at 30°C for 18 h in 6 ml 0.5 M NaOH. After centrifugation at 1,400 g for 5 min, the supernatant was stored and the pellet was washed again with 3 ml 0.5 M NaOH and mixed with the supernatant. The combined alkali extracts were acidified with 1.8 ml concentrated HCl. After precipitation and LTGA recovery by cooling by cooling at 0°C for 4 h and centrifuging at 1,400 g for 5 min and washing two times with 7 ml of distilled water, the pellet was then dried at 60 °C and dissolved in 10 ml 0.5 M NaOH, and the insoluble material was removed by centrifugation. Lignin concentration was measured by reading the absorbance of the supernatant at 280 nm using a Beckman Coulter DU 800 spectrophotometer (Fullerton, CA). Lignin was expressed as mg LTGA/g dry weight.

### Experimental design and statistical analysis

The design of the experiment was completely randomized (CRD) with three replications. Analysis of variance was performed using PROC GLM in SAS (Statistical Analysis System, Copyright 2002–2010, Cary, NC, USA; Windows Version 6.1.7601). *F* and *P*-values are presented in Tables 2, 3. Level of significance was established as such ^*^P ≤ 0.05; ^**^P ≤ 0.01; and ^***^P ≤ 0.001. Means were separated using Fisher's protected Least Significant Difference (LSD) (0.05). Level of significance was ≤0.05 in all measured variables. PROC CORR in SAS was used to establish correlations (*R* and *P*-values) between variables.

**Table 2:** Analysis of variance (*F* and *P*-values) of the effects of year (Y), genotype (G), weathering [(delayed harvest (H)/time of harvest: harvest at harvest maturity (shortly after R8); 14 days after harvest maturity; and 28 days after harvest maturity)], and their interactions for seed protein, oil, fatty acids (%), total boron (TB) (mg kg^−1^), seed coat boron (SCB) (mg kg^−1^), sugars [(sucrose; glucose; and fructose: (mg g^−1^)], germination (Germ) (%), accelerated aging (AA) %, hard-seed (HS) (%), and shattering (Shat) (%) in soybean breeding lines differing in seed germinability under high heat conditions.

		**Protein**		**Oil**		**Palmitic**		**Stearic**		**Oleic**		**Linolenic**	
**Effect**	**DF**	***F***	***P***	***F***	***P***	***F***	***P***	***F***	***P***	***F***	***P***	***F***	***P***
Year (Y)	1	1.5	NS	10.3	^***^	1,159	^***^	260	^***^	98	^***^	410	^***^
Genotype (G)	11	78	^***^	42.6	^***^	13.0	^***^	2.3	^**^	28	^***^	16.9	^***^
Harvest (H)	2	13.2	^***^	12.8	^***^	0.19	NS	11.1	^***^	128	^***^	29.8	^***^
Y × G	11	32.8	^***^	20.9	^***^	2.3	^**^	3.6	^***^	17.5	^***^	20.6	^***^
Y × H	2	6.1	^***^	1.3	NS	0.64	NS	0.36	NS	31.1	^***^	24.9	^***^
G × H	22	4.6	^***^	4.6	^***^	1.6	^*^	0.63	NS	3.3	^***^	7.4	^***^
Y × G × H	21	3.7	^***^	7.6	^***^	2.0	^**^	0.5	NS	8.3	^***^	6.4	^***^
**SEED QUALITY COMPONENT**													
		**TotB**		**SCB**		**Lignin**		**Sucrose**		**Glucose**			
**Effect**	**DF**	***F***	***P***	***F***	***P***	***F***	***P***	***F***	***P***	***F***	***P***		
Year (Y)	1	416	^***^	140	^***^	159	^***^	344	^***^	9.9	^***^		
Genotype (G)	11	41.8	^***^	66.8	^***^	36.0	^***^	7.9	^***^	3.3	^***^		
Harvest (H)	2	926	^***^	234	^***^	8.1	^***^	125	^***^	313	^***^		
Y × G	11	11.6	^***^	2.7	^***^	2.3	^**^	1.7	NS	0.84	NS		
Y × H	2	22.8	^***^	0.68	NS	3.5	^*^	3.7	^*^	28.1	^***^		
G × H	22	6.7	^***^	8.8	^***^	1.3	NS	3.3	^***^	4.8	^***^		
Y × G × H	21	8.1	^***^	7.9	^***^	1.8	^*^	1.2	NS	2.2	^***^		
**SEED QUALITY COMPONENT**													
		**Fructose**		**Germ**		**AA**		**HS**		**Shat**			
**Effect**	**DF**	***F***	***P***	***F***	***P***	***F***	***P***	***F***	***P***	***F***	***P***		
Year (Y)	128	^***^	28.2	^***^	120	^***^	32.8	^***^	1.3	NS	127.7		
Genotype (G)	6.7	^***^	20.9	^***^	21.9	^***^	28.5	^***^	19.6	^***^	6.7		
Harvest (H)	123	^***^	119	^***^	233	^***^	0.84	NS	76.3	^***^	123		
Y × G	4.3	^***^	12.3	^***^	2.1	^*^	32.2	^***^	11.4	^***^	4.3		
Y × H	11.0	^***^	2.6	NS	7.7	^***^	0.37	NS	12.3	^***^	11.0		
G × H	3.3	^***^	2.0	^***^	4.5	^***^	4.8	^***^	7.7	^***^	3.3		
Y × G × H	2.5	^***^	4.3	^***^	6.9	^***^	3.8	^***^	3.7	^***^	2.5		

*Plants were grown in a Sharkey clay soil in 2010 and 2011 at Stoneville, MS, USA*.

* P ≤ 0.05;

** P ≤ 0.01;

*** *P ≤ 0.001; NS, not significant; DF, degree of freedom*.

**Table 3:** Analysis of variance (*F* and *P*-values) of the effects of year (Y), genotypic background (GB) [(two genotypic sources were used: exotic lines and parents and non-exotic (parents and commercial cultivars)], and weathering [(delayed harvest (H)/time of harvest: harvest at harvest maturity (shortly after R8); 14 days after harvest maturity; and 28 days after harvest maturity)], and their interactions for seed protein, oil, fatty acids (%), total boron (TB) (mg kg^−1^), seed coat boron (SCB) (mg kg^−1^), sugars [(sucrose, glucose, and fructose: (mg g^−1^)], germination (Germ) (%), accelerated aging (AA)%, hard-seed (HS) (%), and shattering (Shat) (%) in soybean breeding lines differing in seed germinability under high heat conditions.

		**Protein**		**Oil**		**Palmitic**		**Stearic**		**Oleic**		**Linoleic**		**Linolenic**	
**Effect**	**DF**	***F***	***P***	***F***	***P***	***F***	***P***	***F***	***P***	***F***	***P***	***F***	***P***	***F***	***P***
Year (Y)	1	0.76	NS	4.4	^*^	886	^***^	237	^***^	32	^***^	62	^***^	92	^***^
Genotypic Background (GB)	1	9.6	^***^	4.3	^*^	6.8	^*^	4.8	^*^	9.8	^***^	19.7	^***^	0.76	NS
Harvest(H)	2	0.94	NS	0.31	NS	0.93	NS	0.52	NS	11.2	^***^	19.5	^***^	3.9	^*^
Y × GB	1	9.6	^***^	4.3	^*^	6.8	^*^	4.8	^*^	9.7	^***^	19.7	^***^	0.76	NS
Y × H	2	0.94	NS	0.31	NS	0.93	NS	0.52	NS	11.2	^***^	19.5	^***^	3.9	^*^
GB × H	2	0.15	NS	0.3	NS	3.8	^*^	0.68	NS	0.54	NS	2.2	NS	0.79	NS
Y × GB × H	2	0.15	NS	0.3	NS	3.8	^*^	0.68	NS	0.54	NS	2.2	NS	0.79	NS
**SEED QUALITY COMPONENT**															
		**TB**		**SCB**		**Lignin**		**Sucrose**		**Glucose**					
**Effect**	**DF**	***F***	***P***	***F***	***P***	***F***	***P***	***F***	***P***	***F***	***P***				
Year (Y)	1	113	^***^	41	^***^	77	^***^	236	^***^	7.7	^***^				
Genotypic Background(GB)	1	9.4	^***^	1.3	NS	4.9	^*^	1.2	NS	2.5	NS				
Harvest(H)	2	5.9	^***^	0.12	NS	1.7	NS	2.5	NS	20.2	^***^				
Y × GB	1	9.4	^***^	1.3	NS	4.9	^*^	1.2	NS	2.5	NS				
Y × H	2	5.9	^***^	0.12	NS	1.7	NS	2.5	NS	20.2	^***^				
GB × H	2	1.4	NS	0.44	NS	0.59	NS	0.94	NS	4.1	^*^				
Y × GB × H	2	1.4	NS	0.44	NS	0.59	NS	0.94	NS	4.1	^*^				
**SEED QUALITY COMPONENT**															
		**Fructose**		**Germ**		**AA**		**HS**		**Shat**					
**Effect**	**DF**	***F***	***P***	***F***	***P***	***F***	***P***	***F***	***P***	***F***	***P***				
Year (Y)	1	78	^***^	11.4	^***^	56	^***^	8.1	^***^	0.65	NS				
Genotypic Background(GB)	1	11.6	^***^	7.6	^***^	1.4	NS	3.5	NS	1.3	NS				
Harvest(H)	2	6.7	^***^	0.84	NS	3.4	^*^	0.17	NS	3.9	^*^				
Y × GB	1	11.6	^***^	7.6	^***^	1.4	NS	3.5	NS	1.3	NS				
Y × H	2	6.7	^***^	0.84	NS	3.4	^*^	0.17	NS	3.9	^*^				
GB × H	2	2.5	NS	0.87	NS	3.0	^*^	0.18	NS	0.71	NS				
Y × GB × H	2	2.5	NS	0.87	NS	3.0	^*^	0.18	NS	0.72	NS				

*Plants were grown in a Sharkey clay soil in 2010 and 2011 at Stoneville, MS, USA*.

* P ≤ 0.05;

**P ≤ 0.01;

*** *P ≤ 0.001; NS, not significant; DF, degree of freedom*.

## Results

### Analysis of variance for seed composition and seed quality components

Analysis of variance showed that genotype (G), harvest time (H), and G × Y × H interactions were significant for seed protein and the fatty acids stearic, oleic, and linolenic (Table 2). Similar results for main effect factors and their interactions were observed for other seed composition and quality components. Protein, oil, sucrose, germination, accelerated aging (AA), and shattering were not affected by year, whereas all fatty acids, total and seed coat born, lignin, glucose, fructose, and hardseededness (HS) were all affected by year. In addition, genotypic background (GB) (exotic lines vs non-exotic checks) had significant effects on all seed composition components, except stearic and linolenic acids and glucose (Table 3). There were no significant effects of delayed harvest (H) on some seed composition, such as protein, oil, and palmitic and stearic acids when exotic lines vs non-exotic checks were compared. The interaction between GB and Y was significant for seed composition components, except linolenic acid, SCB, sucrose, and glucose. So, GB × Y interactions had significant effects for the seed composition components protein, oil, palmitic acid, stearic acid, oleic acid, linoleic acid, total boron, lignin, and fructose (Table 3). Except for palmitic acid the Y × GB × H interactions were not significant for seed composition components. For GB × H interactions, only SCB and glucose were significant (Table 3). Genotypic background also had significant effects on total boron (TB) and seed coat B (SCB), sucrose, fructose, and seed quality (germination, hard seed, and accelerated aging) (Table 3). Since the interactions between the main effect factors (G × H × Y) were significant for many seed composition components (Table 2), the results will be presented separately by year as done previously by Bellaloui et al. (2017).

### Effects of delayed harvest on seed protein, fatty acids, total B and seed coat B

Seed protein and oleic acid were higher in the breeding lines with HG (34-3-1-2-4-1, 30-1-4-1-1, 25-1-1-1-1-4, and 24-2-1-2-1-2) than in the non-exotic checks for the non-delayed harvest (H1) in 2010 and 2011 (Tables 4, 5). The HG lines showed different levels of seed composition components in 2010 and 2011(Tables 4, 5). Harvest time showed effects on oleic acid, as it declined for all lines, except LD00-3309 between H2 and H3, after delayed harvests, especially at H3 (28 days after initial harvest). Seed protein decline was observed in only some genotypes. Total B (TB) and seed coat B (SCB) were higher in the HG breeding lines (34-3-1-2-4-1, 30-1-4-1-1, 25-1-1-1-1-4, and 24-2-1-2-1-2) compared to the non-exotic checks, and all parents (PI587982A, DT98-9102, DT97-4290, PI603756, and 5601T) for the H1 harvest in 2010 and 2011 (Tables 4, 5). Generally, TB and SCB decreased in all genotypes after delays in harvest, especially after 28 days of delayed harvest (H3). However, the level of SCB in HG lines was higher after H3 compared with the non-exotic checks (Tables 4, 5).

**Table 4:** Effect of weathering [(delayed harvest (H)/time of harvest: harvest at harvest maturity, shortly after R8 (H1); 14 days after harvest maturity (H2); and 28 days after harvest maturity (H3)] on seed protein (P), oil (O), fatty acids stearic (St), oleic (Ol), linoleic (lin), and linolenic (lino) (%), total boron (TB) (mg kg^−1^), seed coat boron (SCB) (mg kg^−1^), lignin (lig) (%), sugars [(sucrose, Suc; glucose, Glu; and fructose, Fru: (mg g^−1^)], germination (Germ) (%), hard-seed (HS) (%), and shattering (Shat) (%) in soybean breeding lines differing in seed germinability under high heat conditions.

**Genotype**	**P**	**P**	**P**	**O**	**O**	**O**	**St**	**St**	**St**	**Ol**	**Ol**	**Ol**	**Lin**	**Lin**	**Lin**	**Lino**	**Lino**	**Lino**
**HG (Exotic)**	**H1**	**H2**	**H3**	**H1**	**H2**	**H3**	**H1**	**H2**	**H3**	**H1**	**H2**	**H3**	**H1**	**H2**	**H3**	**H1**	**H2**	**H3**
34-3-1-2-4-1	44.6	44.3	43.3	18.9	19.2	18.6	3.5	3.2	3.1	28.9	25.7	24.2	50.1	52.7	54.6	5.6	5.2	5.5
30-1-4-1-1	43.3	43.7	43.1	19.1	18.8	18.9	2.8	2.8	2.8	29.3	25.1	23.0	49.2	51.6	54.2	6.6	6.8	6.6
25-1-1-1-1-4	42.8	42.9	42.5	19.7	19.4	18.7	3.2	2.8	2.9	27.8	25.1	23.2	51.1	53.4	54.5	5.6	5.7	6.1
24-2-1-2-1-2	43.9	42.8	41.3	20.2	20.3	22.3	3.2	3.0	3.3	28.4	24.1	22.4	51.8	54.4	55.1	5.7	5.9	5.6
PI603756	43.8	40.7	42.1	19.1	20.6	22.3	3.2	3.0	3.4	30.1	25.9	23.1	47.4	53.5	55.5	5.5	5.9	5.8
PI587982A	41.4	40.4	41.3	22.8	21.1	21.8	3.5	3.4	3.3	26.0	24.8	21.6	52.1	52.7	57.0	5.8	5.9	6.1
**CHECKS**																		
LD00-3309	42.4	42.7	42.2	19.0	20.1	21.8	3.1	2.6	2.6	22.5	20.7	21.7	56.7	57.5	57.0	6.1	6.4	6.3
DT98-9102	40.9	40.9	41.2	20.6	21.2	20.5	3.5	3.1	3.0	25.7	23.9	21.9	54.3	55.8	56.8	5.6	5.6	5.5
DT97-4290	42.0	40.6	41.2	19.8	21.5	20.2	3.0	2.9	2.8	27.5	23.1	22.1	51.1	55.4	56.3	5.2	6.1	5.8
AG4903	40.1	40.7	40.8	21.7	21.8	21.2	3.2	3.0	3.0	27.6	22.8	20.1	52.2	57.5	58.1	5.5	5.4	5.4
94B73	41.0	40.6	40.6	20.5	21.4	22.1	3.5	3.1	3.1	23.7	22.7	21.0	55.8	55.3	58.4	5.3	5.8	5.5
5601T	41.2	40.6	41.3	20.4	21.1	20.4	3.1	2.8	2.9	23.1	23.0	18.0	56.1	56.0	60.1	5.8	6.3	5.8
LSD	0.40	0.54	0.38	0.40	0.57	0.40	0.20	0.23	0.20	0.70	0.60	0.76	1.10	0.92	0.82	0.30	0.41	0.40
**SEED QUALITY COMPONENT**																		
**Genotype**	**TB**	**TB**	**TB**	**SCB**	**SCB**	**SCB**	**Lig**	**Lig**	**Lig**	**Suc**	**Suc**	**Suc**	**Glu**	**Glu**	**Glu**	**Fruc**	**Fruc**	**Fruc**
**HG (Exotic)**	**H1**	**H2**	**H3**	**H1**	**H2**	**H3**	**H1**	**H2**	**H3**	**H1**	**H2**	**H3**	**H1**	**H2**	**H3**	**H1**	**H2**	**H3**
34-3-1-2-4-1	43.8	37.8	28.9	31.1	28.6	24.7	5.6	5.8	4.7	49.3	45.7	44.0	3.8	3.9	3.2	0.8	0.9	0.8
30-1-4-1-1	42.6	39.0	29.5	29.8	29.3	24.8	6.0	5.1	5.1	45.6	45.3	43.3	3.8	3.8	3.5	0.9	1.0	0.8
25-1-1-1-1-4	42.3	38.6	34.1	30.4	31.2	26.4	4.5	5.2	5.3	45.8	43.0	43.4	4.3	3.9	3.1	1.0	0.9	0.7
24-2-1-2-1-2	42.2	40.0	31.2	29.7	30.9	26.7	6.0	5.5	5.5	50.8	43.7	42.2	4.0	4.5	3.3	1.1	1.2	0.8
PI603756	40.0	37.6	29.2	28.9	26.6	23.9	4.8	4.2	3.4	44.7	43.3	32.8	3.8	3.9	2.7	0.9	1.0	0.6
PI587982A	40.5	38.6	30.3	28.3	26.5	23.8	4.4	3.9	3.5	45.9	40.8	33.7	3.5	4.5	2.4	0.9	1.0	0.6
**CHECKS**																		
LD00-3309	39.6	35.2	28.9	21.8	26.9	21.9	3.0	3.6	3.5	45.3	44.8	33.3	4.0	4.4	2.5	0.9	0.9	0.6
DT98-9102	40.5	35.2	29.8	27.0	26.7	22.3	3.7	3.7	3.4	44.1	45.3	34.4	3.8	4.2	2.5	1.1	0.9	0.6
DT97-4290	41.9	36.8	27.7	26.9	24.3	21.6	3.7	4.1	3.6	47.0	45.8	34.9	3.8	4.7	2.2	0.9	1.2	0.7
AG4903	41.2	36.5	30.0	22.2	27.3	21.1	3.5	3.6	3.3	46.2	46.5	33.3	3.7	4.1	2.3	1.0	1.0	0.6
94B73	39.9	35.1	28.5	25.5	25.0	19.9	3.4	3.9	3.1	43.9	44.6	35.5	3.8	3.9	2.5	0.6	1.0	0.6
5601T	37.5	33.2	27.9	20.6	26.4	20.4	3.2	3.4	3.8	43.8	41.3	34.7	4.4	3.7	2.4	0.9	1.1	0.6
LSD	0.60	0.65	1.11	0.70	0.71	0.76	0.3	0.26	0.30	1.30	0.91	0.69	2.00	0.23	0.21	0.10	0.06	0.03
**SEED QUALITY COMPONENT**																		
**Genotype**	**Germ**	**Germ**	**Germ**	**AA**	**AA**	**AA**	**HS**	**HS**	**HS**									
**HG (Exotic)**	**H1**	**H2**	**H3**	**H1**	**H2**	**H3**	**H1**	**H2**	**H3**									
34-3-1-2-4-1	92.7	71.3	74.0	66.0	32.7	52.7	0.0	0.0	0.7									
30-1-4-1-1	96.0	93.7	67.3	83.0	71.0	14.0	0.3	0.0	0.0									
25-1-1-1-1-4	92.7	72.3	72.0	73.7	39.3	26.7	0.0	0.7	8.3									
24-2-1-2-1-2	83.7	64.0	65.0	68.0	25.7	16.0	0.0	0.3	8.0									
PI603756	86.0	74.3	48.0	93.0	82.0	3.3	5.3	11.7	0.0									
PI587982A	95.7	68.3	32.0	87.0	17.7	2.3	0.3	0.0	0.0									
**CHECKS**																		
LD00-3309	37.3	23.0	11.0	25.0	21.7	19.0	29.3	22.3	25.0									
DT98-9102	88.7	83.3	74.3	48.7	29.0	37.0	0.3	1.0	1.3									
DT97-4290	40.7	12.0	31.7	31.7	10.3	11.7	1.0	1.3	1.3									
AG4903	44.0	39.0	33.3	36.7	16.7	18.7	10.7	9.7	20.3									
94B73	56.7	45.3	22.7	34.3	25.7	6.0	17.7	12.7	0.7									
5601T	75.7	51.7	55.7	40.3	25.3	17.3	0.7	2.3	0.3									
LSD	3.70	4.19	8.75	3.90	4.07	7.75	1.50	0.79	2.30									

*Plants were grown in a Sharkey clay soil in 2010 at Stoneville, MS, USA*.

**Table 5:** Effect of weathering [(delayed harvest (H)/time of harvest: harvest at harvest maturity, shortly after R8 (H1); 14 days after harvest maturity (H2); and 28 days after harvest maturity (H3)] on seed protein (P), oil (O), fatty acids stearic (St), oleic (Ol), linoleic (lin), and linolenic (lino) (%), total boron (TB) (mg kg^−1^), cell wall boron (CWB) (mg kg^−1^), lignin (lig) (%), sugars [(sucrose, Suc; glucose, Glu; and fructose, Fru: (mg g^−1^)], germination (Germ) (%), accelerating aging (AA) (%), hard-seed (HS) (%), and shattering (Shat) (%) in soybean breeding lines differing in seed germinability under high heat conditions.

	**P**			**O**			**St**			**Ol**			**Lin**			**Lino**		
**HG (Exotic)**	**H1**	**H2**	**H3**	**H1**	**H2**	**H3**	**H1**	**H2**	**H3**	**H1**	**H2**	**H3**	**H1**	**H2**	**H3**	**H1**	**H2**	**H3**
34-3-1-2-4-1	43.0	43.5	40.8	19.9	20.0	21.2	4.1	4.1	3.9	29.5	31.0	24.1	50.3	48.9	53.0	6.4	5.9	8.4
30-1-4-1-1	44.8	45.7	43.4	18.0	17.9	20.0	3.8	3.6	3.9	27.3	29.2	30.7	52.0	51.0	49.2	5.3	5.3	6.3
25-1-1-1-1-4	45.5	45.8	44.0	17.9	17.8	17.6	3.8	3.7	3.5	26.5	27.1	25.1	52.8	51.5	51.7	5.7	6.2	8.5
24-2-1-2-1-2	45.9	46.3	46.9	18.0	17.8	17.4	3.8	3.6	3.4	29.0	30.7	26.3	50.7	50.3	51.1	6.2	5.5	8.4
PI603756	41.5	40.2	.	21.5	21.6	.	4.0	3.5	.	20.0	21.5	.	55.6	53.6	.	10.2	10.9	.
PI587982A	41.9	40.7	40.6	20.0	20.0	21.7	3.6	3.5	3.5	27.5	20.4	21.6	51.7	55.5	54.2	5.8	10.2	9.8
**CHECKS**																		
LD00-3309	40.4	40.0	40.8	20.1	20.7	20.1	4.0	3.8	3.5	24.7	26.9	20.9	52.1	52.4	53.8	6.4	6.9	10.6
DT98-9102	40.0	40.7	39.3	21.1	21.2	20.7	3.8	3.8	3.8	25.2	25.0	26.7	54.2	53.7	52.6	8.9	9.1	7.0
DT97-4290	40.3	40.4	40.5	21.5	21.8	21.1	4.1	4.0	3.7	25.5	25.1	24.0	50.8	52.4	54.2	5.6	6.2	8.7
AG4903	43.7	44.1	40.2	19.0	18.9	21.9	3.7	3.5	3.9	25.7	26.9	27.2	52.2	52.9	54.1	7.6	6.3	5.6
94B73	41.0	40.6	42.1	21.9	22.1	18.8	3.9	3.8	3.5	26.4	26.4	24.4	53.3	52.6	52.9	6.2	7.4	8.3
5601T	39.5	39.9	40.8	20.7	21.2	22.4	4.0	3.9	3.7	23.9	24.4	25.7	53.0	53.1	53.1	9.2	8.0	8.0
LSD	0.37	0.30	0.34	0.17	0.20	0.18	0.14	0.06	0.059	0.77	0.62	0.66	0.59	0.60	0.6	0.27	0.28	0.36
**SEED QUALITY COMPONENT**																		
**Genotype**	**TB**	**TB**	**TB**	**CB**	**CB**	**CB**	**Lig**	**Lig**	**Lig**	**Suc**	**Suc**	**Suc**	**Glu**	**Glu**	**Glu**	**Fru**	**Fru**	**Fru**
**HG (Exotic)**	**H1**	**H2**	**H3**	**H1**	**H2**	**H3**	**H1**	**H2**	**H3**	**H1**	**H2**	**H3**	**H1**	**H2**	**H3**	**H1**	**H2**	**H3**
34-3-1-2-4-1	48.5	41.4	36.1	34.6	31.8	26.9	4.7	3.9	4.5	61.6	53.5	43.2	4.0	4.0	3.5	1.2	1.2	1.0
30-1-4-1-1	47.0	41.5	36.0	34.0	31.0	25.6	5.1	4.1	4.2	61.5	56.1	39.5	4.4	4.0	3.5	1.0	1.2	0.9
25-1-1-1-4	48.9	41.1	35.1	33.3	31.2	26.0	4.9	4.2	4.2	62.8	55.6	44.0	3.8	3.9	3.3	1.2	1.3	1.1
24-2-1-2-1-2	46.8	41.9	37.8	33.5	31.1	28.8	4.4	4.1	4.1	57.7	54.2	37.6	4.3	3.9	3.4	1.3	1.4	0.9
PI603756	44.5	41.5	40.1	27.5	31.3	30.7	3.4	2.6	2.8	54.9	54.3	55.0	3.8	3.8	2.3	1.0	1.1	1.0
PI587982A	45.8	39.8	41.5	28.9	28.7	26.8	3.7	2.7	2.8	55.7	54.9	55.6	4.7	3.8	2.4	0.9	1.3	1.0
**CHECKS**																		
LD00-3309	41.0	39.6	36.0	26.6	26.0	23.9	3.2	3.0	3.4	55.1	56.3	56.1	4.7	4.1	2.9	0.9	1.1	0.9
DT98-9102	41.9	40.6	38.9	30.2	25.2	23.8	3.4	3.1	2.5	55.1	55.7	50.5	4.8	3.7	2.6	0.9	0.9	1.0
DT97-4290	43.4	41.1	38.6	26.5	28.5	26.0	3.3	2.7	2.8	60.6	54.5	43.5	4.8	3.9	2.6	0.8	1.2	0.7
AG4903	44.0	37.8	27.0	28.0	29.0	19.2	3.2	2.6	2.7	59.9	53.4	43.3	4.8	3.5	2.4	1.0	1.1	0.7
94B73	43.2	33.1	26.5	27.7	25.1	19.5	3.8	2.6	2.4	56.0	37.9	44.1	4.8	3.8	2.6	1.0	1.0	1.1
5601T	42.2	33.1	26.2	26.6	24.9	19.5	3.5	2.7	2.9	60.1	54.7	43.2	4.8	3.5	3.0	1.0	1.1	1.0
LSD	0.81	0.91	0.62	0.74	0.83	0.58	0.22	0.33	0.24	2.26	4.56	1.49	0.19	0.18	0.19	0.58	0.07	0.058
**SEED QUALITY COMPONENT**																		
**Genotype**	**Germ**	**Germ**	**Germ**	**AA**	**AA**	**AA**	**HS**	**HS**	**HS**									
**HG (Exotic)**	**H1**	**H2**	**H3**	**H1**	**H2**	**H3**	**H1**	**H2**	**H3**									
34-3-1-2-4-1	92.0	78.7	68	90.3	82.3	39.7	0.0	0.0	7.0									
30-1-4-1-1	93.0	83.7	48.3	71.7	45.0	47.3	0.0	0.0	0.0									
25-1-1-1-1-4	91.0	76.0	47.0	61.0	57.3	23.0	1.3	0.0	0.0									
24-2-1-2-1-2	90.0	63.7	.	96.7	75.0	24.0	2.3	2.1	.									
PI603756	95.7	85.5	73.0	84.0	72.7	69.5	0.0	0.5	0.0									
PI587982A	93.0	81.7	59.5	68.0	52.0	27.3	1.3	1.7	0.0									
**CHECKS**																		
LD00-3309	78.0	67.5	38.3	52.0	42.0	26.7	3.3	4.3	0.0									
DT98-9102	78.3	63.0	53.7	48.3	50.7	29.3	8.3	3.7	0.3									
DT97-4290	87.7	62.7	38.3	52.0	38.3	41.3	0.0	0.3	7.0									
AG4903	87.3	61.0	47.0	55.7	40.0	12.0	0.0	4.0	0.0									
94B73	76.3	55.3	45.3	57.3	36.3	25.3	5.7	6.7	0.0									
5601T	81.3	65.3	.	68.0	41.0	32.0	19.3	8.3	6.7									
LSD	3.13	10.8	5.02	4.28	9.94	4.56	0.87	2.13	1.46									

*Plants were grown in a Sharkey clay soil in 2011 at Stoneville, MS, USA*.

*·Data not available due to the delayed harvest effect*.

### Effects of delayed harvest on seed coat lignin and sugars

Lignin was higher in the HG breeding lines (34-3-1-2-4-1, 30-1-4-1-1, 25-1-1-1-1-4, and 24-2-1-2-1-2) compared to the non-exotic checks for all three harvests (H1, H2, and H3) in 2010 and 2011 (Tables 4, 5). Generally, lignin decreased after delays in harvest (H2 and H3) in all genotypes, but the level of lignin content in the HG breeding lines was still higher at each harvest time compared to the non-exotic checks. Levels of sucrose and glucose generally declined with delays in harvest for most lines, whereas fructose levels were similar across times of harvest. Seed sucrose and glucose showed inconsistent differences between HG lines and non-exotic checks across harvest times and years. Breeding lines and other genotypes accumulated different levels of lignin and sugars (Tables 4, 5). A similar pattern was observed when the data were expressed on a genotypic background (exotic line vs. non-exotic check) basis, except that glucose levels did not differ between groups across years (Table 3).

### Effect of delayed harvest on germination, accelerating aging, and hard-seed traits

Germination (Germ) rates, evaluated at initial harvest, were higher in three (34-3-1-2-4-1, 30-1-4-1-1, and 25-1-1-1-1-4) of the HG breeding lines, all derived from PI587982A, compared to the non-exotic checks in both 2010 and 2011 (Tables 4, 5). For AA rate, all exotic lines were higher than all non-exotic lines in 2010 at H1 (Table 4). In 2011, all exotic lines, except 30-1-4-1-1, were higher than all non-exotic lines for AA at H1 (Table 5). Delayed harvesting significantly reduced Germ and AA rates, especially the 28-day delay after initial harvest. Yet in 2010, after a harvest delay of 28 days, 34-3-1-2-4-1 had a higher germination rate (74%) and AA rate (52.7% than any parent or non-exotic line (Table 4). In 2011, after a delayed harvest of 28 days, exotic plant introduction PI603756 had the highest germination rate (73%) and AA rate (69.5%) than any other line (Table 5). No consistent trend was found for the effect of delayed harvest on HS. However, all four HG breeding lines had significantly less HS than cultivars LD00-3309, AG4903, and 94B73 at H1 and H2 in 2010 (Table 4).

### Responses of genotypic backgrounds to delayed harvest for seed quality

**Figure 1:**
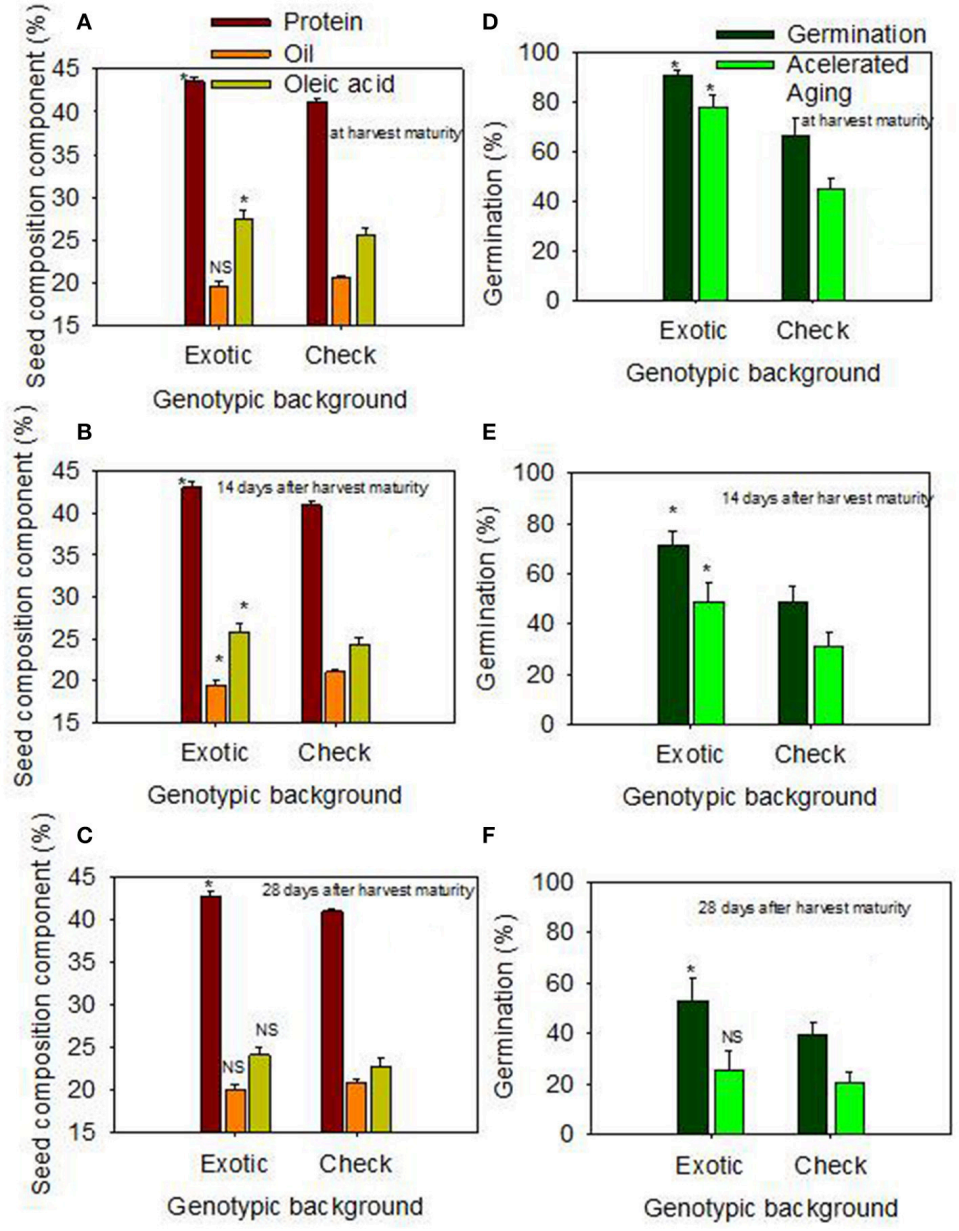
Effect of delayed harvest on seed protein, oil, and oleic acid (%) at harvest maturity **(A)**, 14 days after harvest maturity **(B)**, and 28 days after harvest maturity **(C)**; and effect of delayed harvest on germination and accelerated aging (%) at harvest maturity **(D)**, 14 days after harvest maturity **(E)**, and 28 days after harvest maturity **(F)**. Comparison was made between exotic breeding lines and exotic accessions vs. non-exotic checks (Control): genotypic backgrounds comparison. ^*^ Means there is a significant difference at *P* ≤ 0.05 between similar color bars in each graph. Bars are mean values ± SE. We used the term “harvest maturity” to refer to the time of harvest shortly after full maturity (R8). The experiment was conducted in 2010 and 2011 in Stoneville, MS, USA.

**Figure 2:**
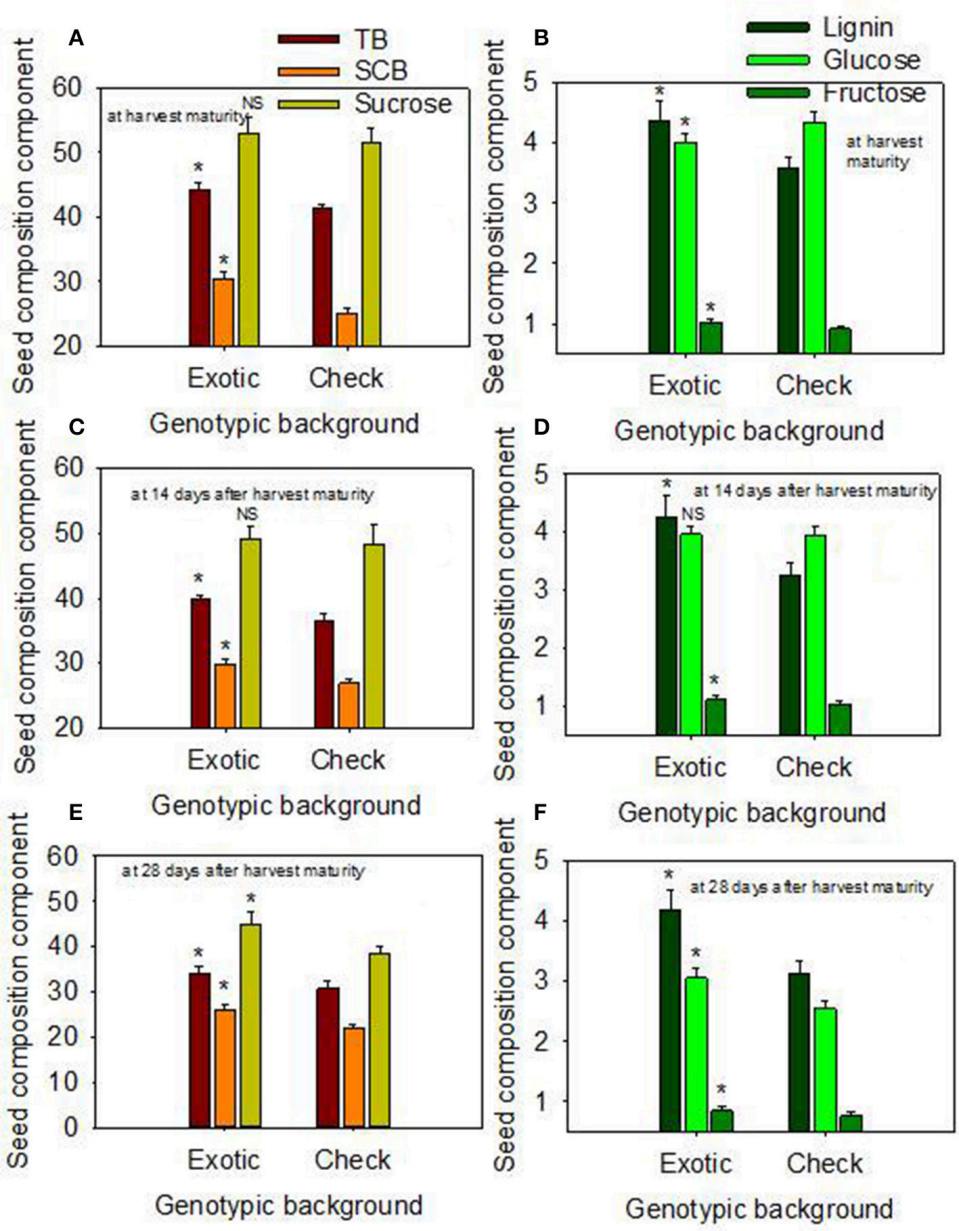
Effect of delayed harvest on total seed boron (TB, mg kg^−1^), seed coat boron (SCB, mg kg^−1^), and seed sucrose (mg g^−1^) at harvest maturity **(A)**, 14 days after harvest maturity **(C)**, and 28 days after harvest maturity **(E)**; and effect of delayed harvest on seed coat lignin [(mg thioglycolic acid (LTGA) g dwt^−1^), seed glucose (mg g^−1^), and seed fructose (mg g^−1^) at harvest maturity **(B)**, 14 days after harvest maturity **(D)**, and 28 days after harvest maturity **(F)**. Comparison was made between exotic breeding lines and exotic accessions vs. non-exotic checks (Control): genotypic backgrounds comparison. ^*^ Means there is a significant difference at *P* ≤ 0.05 between similar color bars in each graph. Bars are mean values ± SE. We used the term “harvest maturity” to refer to the time of harvest shortly after full maturity (R8). The experiment was conducted in 2010 and 2011 in Stoneville, MS, USA.

When the data were expressed based on the two genotypic backgrounds (exotic genotypic background vs. and non-exotic genotypic background), the results showed that seed protein, oleic acid, TB, SCB, Lig, fructose, germination, and accelerated aging were higher in the exotic lines compared to the non-exotic lines (Figure 1). Oil was higher in the non-exotic lines than in the exotic lines. Differences in sucrose were not significant at the H1 or H2 harvests; however, it was higher in exotic breeding lines compared to check varieties at 28 after harvest maturity (Figures 1, 2).

### Correlations between germination and seed composition and seed quality component traits

There were significant positive correlations between germination and protein, stearic, oleic, TB, SCB, and sugars (sucrose, glucose, and fructose) (Table 6). However, negative correlations were observed between germination and yield, oil, linoleic fatty acid, hard-seed, and seed wrinkling. These correlation were obtained when all variables of seed composition components and seed quality components were correlated across all genotypes, harvesting times (H1, H2, and H3), and years. This approach will provide us with a more accurate pattern of changes across the entire genotypic spectrum. These correlations include 216 combined variables. Therefore, a low value of R, such as ±0.2 can represent a highly significant correlation (^***^) between two variables.

**Table 6:** Correlation (coefficient of correlation, *R*; and significant level, *P*) between germination (Germ) and accelerating aging (AA), and seed composition components (protein, P; oil, O; palmitic, Pal; stearic, St; oleic, Ol; linoleic, Lin; linolenic, Lino; hard-seed, HS; shattering, Shat; yield; total boron, TB; cell wall boron, CWB; lignin, Lig; sucrose, Suc; glucose, Glu; and fructose, Fru across years and across genotypes and across three harvesting times (harvest at harvest maturity, shortly after R8 (H1); 14 days after harvest maturity (H2); and 28 days after harvest maturity (H3) in soybean breeding lines differing in seed germinability under high heat conditions. Six exotic breeding lines and accessions and six non-exotic checks were used.

	**P**	**O**	**Pal**	**St**	**Ol**	**Lin**	**Lino**	**Germ**	**AA**	**HS**	**Shat**	**Yield**	**TB**	**CWB**	**Lig**	**Suc**	**Glu**	**Fru**
Germ	*R* = 0.5	−0.5	−0.0	0.3	0.5	−0.5	−0.0	1.0	0.7	−0.5	−0.1	−0.4	0.6	0.5	0.3	0.5	0.4	0.3
	*P* = ^***^	^***^	NS	^***^	^***^	^***^	NS	NA	^***^	^***^	NS	^***^	^***^	^***^	^***^	^***^	^***^	^***^
AA	*R* = 0.3	−0.3	−0.2	0.3	0.6	−0.6	0.03	0.7	1.0	−0.2	−0.2	−0.4	0.6	0.6	0.19	0.5	0.4	0.4
	*P* = ^***^	^***^	^*^	^***^	^***^	^***^	NS	^***^	N	^***^	^*^	^***^	^***^	^***^	NS	^***^	^***^	^***^
HS	*R* = −0.2	0.2	0.0	−0.2	−0.3	0.3	0.0	−0.5	−0.2	1.0	−0.1	0.2	−0.1	−0.3	−0.1	−0.2	0.01	−0.2
	*P* = ^*^	^*^	NS	^**^	^***^	^***^	NS	^***^	^***^	NA	^*^	NS	NS	^***^	NS	^*^	NS	^*^
Shat	*R* = 0.3	−0.2	0.2	−0.1	−0.0	0.0	−0.1	−0.10	−0.2	−0.1	1.00	−0.3	−0.2	−0.03	0.1	−0.2	−0.3	−0.1
	*P* = ^***^	^***^	^*^	NS	NS	NS	NS	NS	^*^	^*^	NA	^*^	^***^	NS	NS	^*^	^***^	NS
Wr	*R* = −0.5	0.21	−0.2	−0.1	−0.4	0.4	0.2	−0.74	−0.7	0.6	−0.5	0.3	−0.4	−0.7	−0.3	−0.2	0.2	−0.1
	*P* = ^***^	NS	NS	NS	^***^	^***^	NS	^***^	^***^	^***^	^***^	^*^	^***^	^***^	^**^	NS	NS	NS
Yield	*R* = −0.1	0.2	0.7	−0.6	−0.0	0.1	−0.4	−0.4	−0.4	0.2	−0.3	1.00	−0.6	−0.3	0.4	−0.8	−0.5	−0.2
	*P* = NS	NS	^***^	^***^	NS	NS	^***^	^***^	^***^	NS	^*^	NA	^***^	^**^	^***^	^***^	^***^	NS
TB	*R* = 0.3	−0.3	−0.2	0.4	0.5	−0.5	0.1	0.6	0.7	−0.1	−0.2	−0.6	1.0	0.8	0.1	0.7	0.6	0.5
	*P* = ^***^	^***^	^***^	^***^	^***^	^***^	NS	^***^	^***^	NS	^***^	^***^	NA	^***^	NS	^***^	^***^	^***^
CWB	*R* = 0.5	−0.4	−0.1	0.2	0.5	−0.5	0.0	0.5	0.6	−0.3	−0.0	−0.3	0.8	1.00	0.3	0.6	0.4	0.5
	*P* = ^***^	^***^	NS	^***^	^***^	^***^	NS	^***^	^***^	^***^	NS	^*^	^***^	NA	^***^	^***^	^***^	^***^
Lig	*R* = 0.4	−0.3	0.6	−0.3	0.2	−0.2	−0.3	0.3	0.1	−0.1	0.1	0.4	0.1	0.3	1.0	0.0	0.2	0.0
	*P* = ^***^	^***^	^***^	^***^	^**^	^**^	^***^	^***^	NS	NS	NS	^***^	NS	^***^	NA	NS	^**^	NS
Suc	*R* = 0.3	−0.3	−0.5	0.5	0.5	−0.5	0.2	0.5	0.5	−0.2	−0.2	−0.8	0.7	0.6	0.0	1.0	0.6	0.5
	*P* = ^***^	^***^	^***^	^***^	^***^	^***^	^***^	^***^	^***^	^*^	^*^	^***^	^***^	^***^	NS		^***^	^***^
Glu	*R* = 0.1	−0.1	−0.1	0.2	0.4	−0.3	−0.1	0.4	0.4	0.0	−0.3	−0.5	0.6	0.4	0.2	0.6	1.00	0.4
	*P* = NS	^*^	NS	^***^	^***^	^***^	NS	^***^	^***^	NS	^***^	^***^	^***^	^***^	^***^	^***^	N/A	^***^
Fru	*R* = 0.3	−0.3	−0.3	0.3	0.4	−0.4	0.2	0.3	0.4	−0.2	−0.1	−0.2	0.5	0.5	0.00	0.5	0.4	1.00
	*P* = ^***^	^***^	^***^	^***^	^***^	^***^	^*^	^***^	^***^	^**^	NS	NS	^***^	^***^	^***^	^***^	^***^	NA

*Plants were grown in a Sharkey clay soil in 2010 and 2011 at Stoneville, MS, USA*.

* P ≤ 0.05;

** P ≤ 0.01;

*** *P ≤ 0.001; NS, not significant; NA, not applicable*.

## Discussion

### Effects of year, genotype, harvesting time, and their interactions

**Figure 3:**
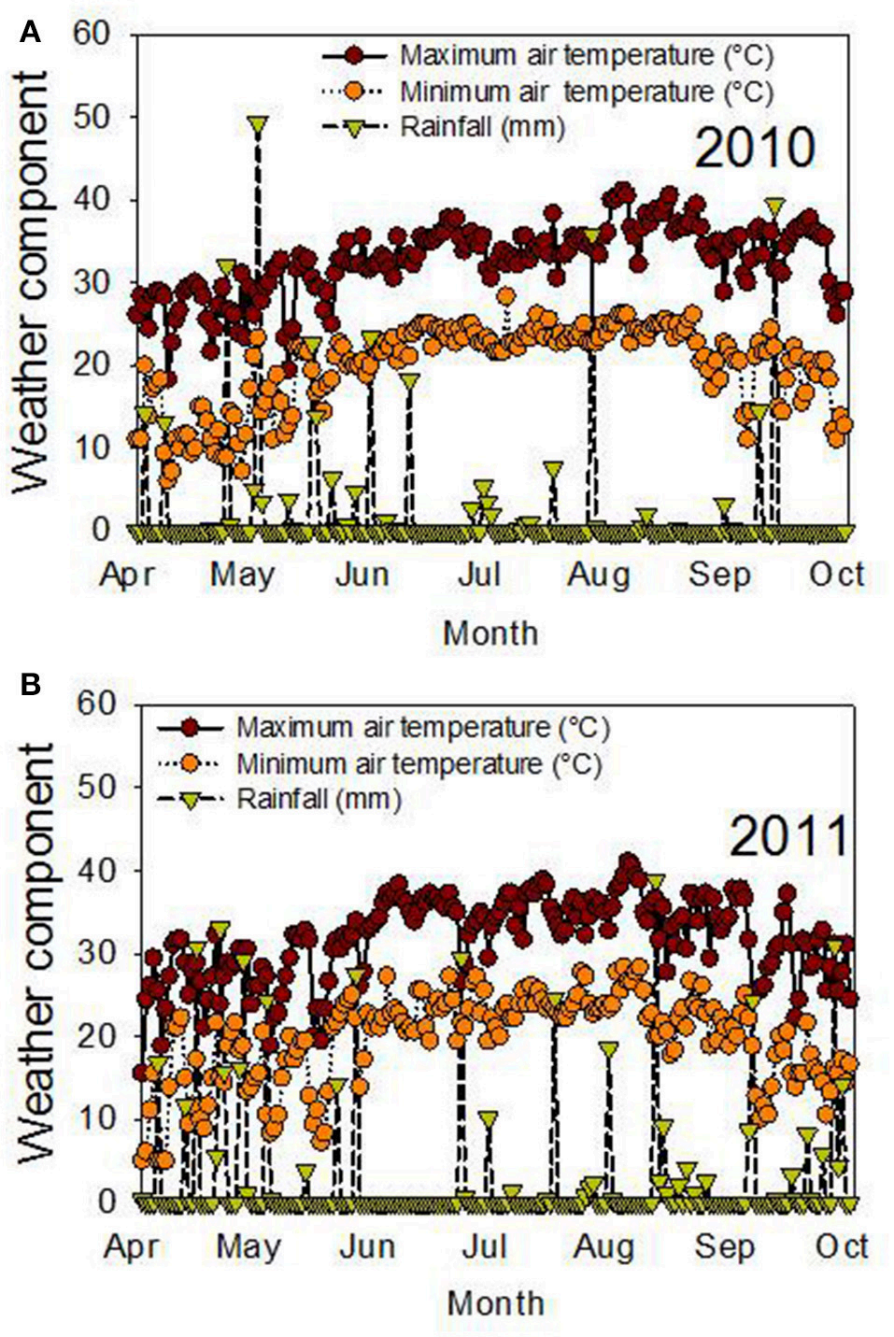
Air temperature (°C) and rainfall (mm) during the growing season in 2010 **(A)** and 2011 **(B)**. The experiment was conducted in 2010 and 2011 in Stoneville, MS, USA.

The significant effects of genotype (G), time of harvest (H), and interactions of G × Y × H for protein and fatty acids indicated that these factors are the main driving force behind the differences in these seed nutritional components. The significant interactions of these factors for these constituents indicated that the environmental differences in each year affected these constituents differently, and the response of these constituents at each harvest time was also different. Since the experiment was irrigated, the differences in each year could be due to temperature (Figure 3). For example, weather data (MSUCares, 2017) indicate that the maximum temperature in April was 30°C in 2010 and 28°C in 2011. In August, the maximum temperature was 37°C in 2010 and 35.4°C in 2011. These differences in temperature, especially in August when seed-fill occurs, could be a source of seed quality component differences in each year. Previous research showed that different patterns of temperature in each year affected seed composition differently (Bellaloui et al., 2009, 2017). The non-significant effect of year (Y) for most of the seed quality components indicates that the pattern of these components had a similar effect in each year. The significant effect of genotypic background (GB), Y, H, and their interactions, when the data were analyzed based on exotic line vs. non-exotic check backgrounds, indicated that the differences in seed quality components of the seed were due to these factors. The interaction effects of these factors for seed quality components indicated that the differences in these components changed each year and that they were probably due to the temperature differences in each year. The response of these components to harvest time depended on each genotypic background, whether exotic or non-exotic.

### Effects of delayed harvest on seed protein, fatty acids, seed coat B and lignin, and sugars

A recent study evaluated 16 soybean genotypes, among them four breeding lines expressing the HG trait under high heat environments, and showed that these HG lines had high seed protein and oleic acid (Bellaloui et al., 2017). The association between seed protein or oleic acid with germination is still not clear. LeVan et al. (2008) investigated the influence of seed composition and germination under a controlled environment and under field conditions. They concluded that seed composition components, such as protein, oil, and fatty acids, may play a major role in imbibitional injury at low seed moisture content. They also observed a quadratic relationship between seed protein content and seed germination, but suggested further research was needed on the relationship between seed germination and protein, and fatty acids. Recently, Chebrolu et al. (2016) observed low protein synthesis or protein degradation in the heat sensitive genotype DT97-4290 (a non-exotic check in the current study) under high heat compared to the heat tolerant genotype 25-1-1-4-1-1 (a sister line of 25-1-1-1-1-4 in the current study), which maintained similar protein levels across multiple temperature environments (28/22°C, 36/24°C, and 42/26°C day/night). A previous study (Bellaloui et al., 2017) involving high field temperatures showed that breeding line 25-1-1-4-1-1 showed higher protein content (43.3 and 40.1% for 2012 and 2013, respectively) than cultivar AG4903 (38.9 and 37.2%, respectively. AG4903 is also included in the current study. These observations support the current study, where protein levels of AG4903 were less than those of HG line 25-1-1-1-1-4 in both 2010 and 2011 and strengthen the notion that HG lines have higher protein levels than lines from the traditional germplasm pool under high heat conditions. The high levels of total B and seed coat B and lignin in the seed coats of HG lines could be due to the role of B and lignin in germination and seed coat protection. It is well established that B has structural and metabolic roles, being involved in cell wall structure and cell wall and cell membrane integrity. Boron has a role in protecting the seed coat from deterioration by preventing cell wall, cell membrane, and seed coat leaking. A recent study showed significantly higher accumulation of K, P, N, B, Cu, and Mo in seeds of the two HG/heat tolerant breeding lines 25-1-1-4-1-1 and 34-3-1-2-4-1, when compared to the other 14 genotypes grown under the high heat of the Mississippi Delta (Bellaloui et al., 2017). HG breeding line 34-3-1-2-4-1 was also included in the current study. This suggests that these nutrients, including B, may play important roles in seed germination. Boron has been considered one of the specific nutrients, in addition to Ca, K, Cu, Fe, Co, Zn, and Mn, involved in seed coat resistance, phenol metabolism, and lignin biosynthesis, which are involved in membrane permeability and integrity (Marschner, 1995). To our knowledge this is the first report of relating B and lignin levels in the seed coat to germination rates under high heat conditions. Additional research is needed to further clarify these relationships.

The role of seed coat lignin (lig) for seed composition, germination, vigor, and seed and seed coat physical and chemical properties was previously reported by Pereira et al. (1985), Alvarez et al. (1997), Romkaew et al. (2008), Bellaloui et al. (2012b), Baldoni et al. (2013), and de Oliveira et al. (2014). However, in these studies, there were no lines with HG. Therefore, the objective of this research was to advance our knowledge of boron nutrition and further understand the relationships between seed coat B and Lig to seed nutrition and seed germinability under high heat conditions using HG lines. Our results show that all HG lines had high Lig at full maturity, but the content of Lig declined with delays in harvest even in the HG lines, being the lowest at 28 days after initial harvest. However, the content of Lig in the HG lines was significantly higher than that in the non-exotic checks, even after 28 days from initial harvest, indicating that Lig could be associated with seed germination and health of the seed. Seed coat lignin in HG lines was accompanied by significantly less seed coat wrinkling at the H1 harvest compared with the non-exotic checks, even at 28 days after initial harvest. Seed coat lignin content was different between lines due to genotypic differences.

Although seed sugars did not show clear differences between HG lines and non-exotic checks at H1, higher levels of sucrose, glucose, and fructose were shown in HG lines than in the non-exotic checks at delayed harvest as well. This indicates that HG lines maintained higher levels of sugars under stress environments (weathering effects), which may be due to less leakage of these sugars through the seed coat and seed membrane as a result of the higher content of SCB and Lig. Both Lig and SCB were reported to be involved in carbohydrate and phenolic metabolism (Marschner, 1995) to protect cell walls and cell membranes from the physical damage of seed coat deterioration (Pereira et al., 1985; Alvarez et al., 1997; Romkaew et al., 2008; Bellaloui et al., 2012b; Baldoni et al., 2013; de Oliveira et al., 2014), especially under stress environments. The biological functions of the oligosaccharides are not clear (Ren et al., 2009), but other researchers, for example, Obendorf (1997) reported that oligosaccharides and sucrose are important for desiccation tolerance during soybean seed development and maturation, and protection of seeds against damage during seed dehydration and aging. It should be noted that published research has shown that the activity of sucrose synthase in nodules, the main enzyme involved in sucrose hydrolysis, decreased under conditions of drought (González et al., 1995; Streeter, 2003), suggesting higher sensitivity of sucrose to abiotic stress.

### Effect of delayed harvests on germination, accelerating aging, and hard-seed traits

The higher Germ and AA in HG lines is expected as these lines were bred for HG. The lower HS rates are also expected as germination is inversely correlated with HS. Previous research showed that high germinating genotypes had the lowest hard seed and seed wrinkling percentages (Smith et al., 2008; Mengistu et al., 2009, 2010; Bellaloui et al., 2012c, 2017). Smith et al. (2008) evaluated 486 accessions, 24 ancestors, and cultivars Stalwart, Croton 3.9, and Stressland with MG ranging from 000 to MG V under field conditions under the ESPS of the midsouthern USA in 2002 and 2003, and a significant negative correlations was observed between standard germination and hard seed, and wrinkled seed, phomopsis, and seed weight. Mengistu et al. (2009) evaluated genotypes under irrigated and non-irrigated conditions and reported that genotypes with higher germination showed lower hard-seed rate and lower phomopsis seed infection. It was earlier reported that high temperatures with alternating periods of wet and dry conditions, such as in the ESPS of the Midsouth, lead to lower germination and increased seed coat wrinkling (Franca-Neto et al., 1993). Similar results were found for seed germination and hard seed by Bellaloui et al. (2012c). Although the cause of poor seed quality in the ESPS is still not fully understood, high temperature, soil moisture, and disease infection, especially by *Phomopsis*, during seed-fill and pre-harvest lead to hard-seed and low seed viability and vigor (TeKrony et al., 1980; Roy et al., 1994). The differences in Germ, AA, and HS among the genotypes are due to genotypic differences and environmental conditions that vary each year (Smith et al., 2008; Mengistu et al., 2009; Bellaloui et al., 2017).

### Responses of genotypic backgrounds to delayed harvest for seed quality

The higher contents of protein, oleic acid, TB, SCB, Lig, fructose, and the higher rates Germ, and AA in HG lines with exotic genotypic backgrounds confirm our hypothesis that these seed nutritional qualities and seed physical and chemical properties of the seed coat could be associated with high Germ and AA in these lines as shown in Figures 1, 2. The higher oil in the non-exotic check background than in the exotic background is due to the inverse relationship between protein and oil (Burton, 1985; Wilcox and Shibles, 2001; Bellaloui et al., 2009). In our analysis, we used two models of statistical analyses. One model was based on the contribution of each genotypes to the variability of the variable studied (seed quality components); the other model was based on the contribution of two categories (exotic vs non-exotic) (genotypic background, GB) to the variability of the seed quality components. Consequently, there were no significant effects of delayed harvest (H) on some seed composition, such as protein, oil, and palmitic and stearic acids when exotic lines vs non-exotic checks were compared, resulting in inconsistent effects of delayed harvest on some seed composition constituents. This may be due to the fact that there must be enough significant variance among individual genotypes to result for an overall significant effect of delayed harvest for some seed constituents. However, when the lines are grouped into only 2 categories (exotic vs non-exotic), the diversity is minimized by comparing only the 2 means.

### Correlations between germination and seed composition and seed quality components

The significant positive correlations between Germ and seed quality components [(protein, stearic, oleic, TB, SCB, and sugars (sucrose, glucose, and fructose)] support our previous observation that these components are involved in germination. Previous research reported that a quadratic relationship between seed protein content and seed germination existed, but the relationship between germination and oil was not conclusive (LeVan et al., 2008). They concluded that further research was needed to better understand the relationship between germination and fatty acids. Also, previous research showed that HG was significantly correlated with seed soluble and structural B (Bellaloui et al., 2009, 2012c, 2017). The negative correlation between germination and HS (Table 6) was also expected as germination and HS are generally inversely correlated because seed that cannot imbibe water cannot germinate (Smith et al., 2008; Mengistu et al., 2009; Bellaloui et al., 2017).

## Conclusion

The current research confirmed that selecting soybean lines with HG and heat tolerance resulted in lines with higher protein, oleic acid, sugars, and seed coat B and seed coat lignin, confirming our hypothesis. Higher protein and oleic acid are desirable traits for high soymeal quality and oil stability for shelf-life and biodiesel production. High levels of sucrose, glucose, and fructose are desirable for taste and add extra calories to soymeal, such that fewer caloric additives are necessary. Since HG lines had higher seed coat boron and seed coat lignin even at delayed harvest, involvement of seed coat B and seed coat lignin with the HG trait could be a possibility and needs further research. Time of harvest is a major management factor affecting, not only seed production, but also seed nutrition and quality. Both breeding lines 25-1-1-1-4 and 24-2-1-2-1-2 showed competitive high yield in addition to their high seed quality traits.

## Author contributions

NB contributed to the planning, analysis, interpretation, and writing. JS conducted the field portion of the experiment and contributed to the planning, data interpretation, and writing. AM contributed to the analysis, data interpretation, and writing.

### Conflict of interest statement

The authors declare that the research was conducted in the absence of any commercial or financial relationships that could be construed as a potential conflict of interest.
